# Sequential Proteomic Analysis Reveals the Key APOE4‐Induced Pathological and Molecular Features at the Presymptomatic Stage in Alzheimer's Disease Mice

**DOI:** 10.1111/cns.70306

**Published:** 2025-03-12

**Authors:** Pengju Wei, Kaihua Lin, Xuhui Chen, Cheng Fang, Linhui Qiu, Jun Hu, Junlei Chang

**Affiliations:** ^1^ State Key Laboratory of Biomedical Imaging Science and System Institute of Biomedicine and Biotechnology, Shenzhen Institute of Advanced Technology, Chinese Academy of Sciences Shenzhen Guangdong China; ^2^ Shantou University Medical College Shantou Guangdong China; ^3^ Department of Neurology Peking University Shenzhen Hospital Shenzhen Guangdong China

**Keywords:** Alzheimer's disease, APOE4, mitochondrial dysfunction, neuroinflammation, proteomics, synaptic degeneration

## Abstract

**Aims:**

Alzheimer's disease (AD) involves a prolonged presymptomatic or preclinical stage with subtle pathological changes. Apolipoprotein E4 (APOE4) is a significant genetic risk factor for AD, yet its specific role at the presymptomatic stage is not fully understood. This study aimed to elucidate the cellular and molecular effects of APOE4 compared to APOE3 on AD progression during the presymptomatic stage.

**Methods:**

We generated *5xFAD* AD mice carrying human APOE3 or APOE4 and their non‐AD controls. Behavioral tests, immunostaining, quantitative proteomics and phosphoproteomics, Golgi staining, and Western blotting were conducted at 3 or 10 months of age, respectively. Cell culture experiments were performed to assess APOE4's direct impact on neuronal mitochondrial function.

**Results:**

APOE4 significantly increased β‐amyloid (Aβ) deposition and microglial activation compared to APOE3 in *5xFAD* mice at the presymptomatic stage, without aggravating the blood–brain barrier disruption. Proteomic and biochemical analysis revealed strong molecular features of synaptic degeneration and mitochondrial dysfunction associated with APOE4. Notably, APOE4 promoted mitochondrial fusion and mitophagy while inhibiting fission, leading to impaired neuronal energy supply and increased reactive oxygen species.

**Conclusion:**

Our findings indicate that APOE4 accelerates AD pathologies at the presymptomatic stage by exacerbating Aβ deposition, neuroinflammation, and synaptic degeneration. The study highlights mitochondrial dysfunction as a critical mediator of APOE4‐induced AD progression, providing potential targets for early intervention.

## Graphical abstract

This study demonstrates that compared to APOE3, APOE4 exacerbates Aβ deposition, microglial activation, and synaptic degeneration in the presymptomatic stage of *5xFAD* mice. Proteomics and phosphoproteomics reveal the potential mitochondrial mechanisms by which APOE4 promotes synaptic degeneration.

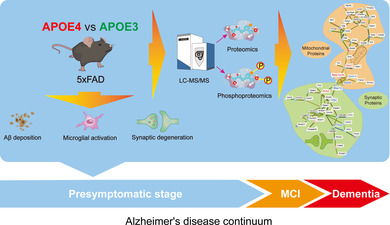

## 1 Introduction

Alzheimer's disease (AD) is the most common cause of dementia. It is estimated that the global number of people with AD, including the prodromal cognitive impairment stage, is more than 400 million [1]. The diagnosis of AD typically begins with a thorough clinical assessment to evaluate memory, language, and other cognitive functions, followed by an assessment of cerebrospinal fluid (CSF) and blood biomarkers, genetic testing, and brain imaging [2]. According to the 2018 NIA‐AA ATN research framework, β‐amyloid (Aβ) deposition (A), pathologic tau (T), and neurodegeneration (N) are the major hallmarks of AD [3]. Despite recent advances in the development of disease‐modifying therapies that target the underlying pathophysiology of AD, for instance, monoclonal antibodies aiming to reduce Aβ plaques in the brain, there remains a huge unmet clinical need for the treatment of AD, especially for the early intervention of AD [4]. Although sharing similar cognitive impairments with other types of dementia, AD now emphasizes a progressive onset process over decades, from cognitively normal (presymptomatic or preclinical stage) to mild cognitive impairment (prodromal stage) and final dementia, a trajectory termed the AD continuum [5]. The pathological changes at the molecular level in the AD brain occur much earlier than the emergence of clinical symptoms. For example, brain Aβ deposition and increased concentrations of tau protein in the CSF can be detected 15 years before expected symptom onset in patients with autosomal dominant AD [6]. It is currently believed that the lengthy presymptomatic or preclinical stage of AD is a critical period for early intervention, during which neurodegeneration has not yet progressed to an irreversible extent [7, 8].

Synaptic connections are widely acknowledged as the structural basis for cognitive functions, and their degeneration is linked to cognitive impairments in numerous neurodegenerative diseases [9]. In AD, synaptic degeneration initiates during the preclinical stages and exacerbates as cognitive decline progresses [10]. Furthermore, among the various pathological changes in AD, the loss of synapses correlates most significantly with the deterioration of cognitive function [11]. The reduction of dendritic spines observed in an AD animal model provides clear evidence of synaptic loss [12]. The Aβ hypothesis posits that Aβ is a central element in the pathogenesis of AD, a concept that is bolstered by substantial evidence, including the demonstrated direct toxic effects of Aβ oligomers on synapse [13]. Neurons are among the most energy‐intensive cells in the body, relying heavily on ATP generated by mitochondria for their fundamental physiological activities and the execution of neural functions. Mitochondrial dysfunction has also been shown to be a significant pathological change in the early stages of AD, even before severe synaptic damage [14]. Aβ impairs mitochondrial function and affects the recruitment of mitochondria to synapses [15]. Increasing evidence suggests mitochondrial dysfunction plays a crucial role in synaptic degeneration during the early stages of AD [16, 17]. However, the molecular mechanisms driving mitochondrial dysfunction and synaptic degeneration at the presymptomatic stage of AD are not yet fully understood.

Human Apolipoprotein E (APOE) protein has three major isoforms: APOE2, APOE3, APOE4. APOE3 is the most prevalent isoform, differing from APOE4 and APOE2 by a single amino acid at position 112 or 158, respectively [18]. The risk of AD increases in a gene dose‐dependent manner from APOE2 to APOE4, and APOE4 is associated with a lower age at onset of AD [19, 20]. The APOE ε4 allele was traditionally viewed as the strongest genetic risk factor for sporadic AD [21]. But a recent large multicenter cohort study involving more than 10,000 participants demonstrated a near‐full penetrance of AD biology in APOE ε4 homozygotes, suggesting that APOE ε4 homozygosity represents a distinct genetic form of AD, rather than just a risk factor [22]. Previous studies found that APOE4 is extensively involved in the pathological processes related to AD, including Aβ deposition, synaptic degeneration, mitochondrial dysfunction, neuroinflammation, blood–brain barrier (BBB) disruption and other processes [23]. The extensive involvement of APOE4 in AD has led some scholars to propose the “APOE Cascade Hypothesis” to address the impact of APOE4 on AD [24, 25]. However, what pathological processes are induced by APOE4 at the presymptomatic stage of AD, the occurrence order of these processes, and related molecular mechanisms remain unclear.

In this study, we developed *5xFAD* AD mice carrying human APOE3 or APOE4, along with their non‐AD controls, to investigate the impact of APOE4 compared to APOE3 on AD progression during the presymptomatic stage, focusing on cognitive function, Aβ deposition, microglial activation, and BBB permeability. Additionally, we utilized Label‐free quantitative proteomics to analyze the proteome and phosphoproteome in the hippocampus of these mice at 3 and 10 months of age, respectively, aiming to elucidate the underlying molecular mechanisms. The results revealed significant molecular features of synaptic degeneration and mitochondrial dysfunction induced by APOE4 at the presymptomatic stage of AD. These findings enhance our understanding of the molecular mechanisms driving APOE4's pathogenic role in AD and identify new therapeutic targets for further functional and pharmacological investigation.

## 2 Material and Methods

### 2.1 Animals

The humanized *APOE4* (*APOE4* ^*+/+*^, Cat. #NM‐HU‐190002) and *APOE3* (*APOE3* ^*+/+*^, Cat. #NM‐HU‐190003) knock‐in mice on C57BL/6J background were purchased from Shanghai Model Organisms Center Inc. In both strains, the endogenous mouse *Apoe* gene was replaced by either human *APOE4* or *APOE3* gene. The *5xFAD* AD transgenic mouse model (Jackson Laboratory, MMRRC Strain #034848‐JAX, Tg6799, C57BL/6J background), which overexpresses both mutant human amyloid beta precursor protein 695 (APP) with the Swedish (K670N, M671L), Florida (I716V), and London (V717I) Familial Alzheimer's Disease (FAD) mutations and human presenilin 1 protein (PS1) with two FAD mutations (M146L, L286V) under the control of neural‐specific *Thy1* promotor [26], was used in this study. Homozygous *APOE4* (*APOE4* ^*+/+*^; *5xFAD* ^*−/−*^, termed as E4‐wild type (WT) mice) or *APOE3* (*APOE3* ^*+/+*^; *5xFAD* ^*−/−*^, termed as E3‐WT mice) knock‐in mice were crossed with heterozygous *5xFAD* transgenic mice to generate mice co‐expressing human APOE4, APP, and PS1 (*APOE4* ^*+/+*^; *5xFAD* ^*+/−*^, termed as E4‐AD mice) or mice co‐expressing human APOE3, APP, and PS1 (*APOE3* ^*+/+*^; *5xFAD* ^*+/−*^, termed as E3‐AD mice). Only male mice were used to keep consistency and reduce intra‐group variations in the behavior tests. Animals were housed in 12‐h light and dark cycles in a pathogen free animal facility with free access to food and water. All animal experiments were performed following the “Principles of laboratory animal care” (NIH publication No. 86–23, revised 1985) and in accordance with the National Institutes of Health guidelines for use and care of live animals, and were approved by the Institutional Animal Care and Use Committee of Shenzhen Institute of Advanced Technology, Chinese Academy of Sciences.

### 2.2 Open Field Test

Open field test was conducted using a 40 cm × 40 cm × 40 cm cubic box that was uncovered. The bottom of the box was evenly divided into 16 square areas. The 4 central squares were defined as the center zone, while the remaining 12 squares were defined as the corner zone. Mice were allowed to move freely within the box for 5 min. A digital camera was placed directly above the box to record the movements of the mice. The Video tracking software SMART V3.0 (Planlab) was used to analyze the speed, distance, and time of the mice's movements, and to generate track graphs in all behavior tests.

### 2.3 Y‐Maze Spontaneous Alternation Test

The Y‐maze spontaneous alternation test was performed using a Y‐maze consisting of three arms, each 35 cm long and 10 cm wide, with walls 15 cm high. Mice were placed into the Y‐maze at the end of one arm and allowed to move freely for 8 min. The transitions between arms were recorded in sequence for analysis. A correct spontaneous alternation was defined by no repeated entrance in any set of 3 successive arm transitions. Each arm transition, along with the two preceding transitions, constituted a condition for determining spontaneous alternation. The percentage of correct spontaneous alternation was calculated by the total number of correct spontaneous alternation/(total arm entrances‐2) × 100, as described previously [27].

### 2.4 Novel Object Recognition Test

The novel object recognition (NOR) test was conducted using the same cubic box as the open field test. The NOR test took 3 days to be completed. On the first day, the mice were placed in the box to acclimate for 10 min. On the second day, two white plastic cubes of the same size were added to the box. Mice were allowed to explore freely for 10 min in the box to become familiar with the objects. On the final day, one of the cubes was replaced with a blue plastic cone, which served as the novel object. 5‐minute (min) free movements of mice in the box were recorded. Recognition of the objects was defined as mice making contact with them using their noses. Mice with a total recognition time of less than 6 s were excluded from the final data analysis. The NOR index was calculated as recognition time of the novel object/total recognition time × 100.

### 2.5 Morris Water Maze Test

The Morris water maze test was conducted as previously described [28]. In brief, the test was performed in a circular pool with a diameter of 120 cm, which was filled with water. The water temperature was kept between 18°C and 20°C. The surface of the water was evenly divided into four sector areas, which were defined as quadrants I, II, III, and IV in a clockwise direction, sequentially. A platform was placed under water in the center of quadrant IV (target quadrant). Food‐grade titanium dioxide was mixed in the water to make the platform invisible. Mice received 5 days of training for learning to find the platform. On the sixth day, the platform was removed. Mice underwent a 60‐s probe trial to test their memory.

### 2.6 Immunofluorescence Staining

After cardiac perfusion with cold PBS, the mouse brains were harvested and sectioned into 2‐mm‐thick coronal slices using a brain matrix. The hippocampus‐containing brain slices were collected and fixed in 4% Paraformaldehyde at room temperature for 1 h. They were then transferred into 15% and 30% sucrose solutions at 4°C overnight for gradient dehydration. 10‐μm‐thick coronal brain slices were made using a freezing microtome (Leica, CM1950). The brain slices were washed with PBS to remove OCT. After blocking with a buffer containing 5% goat serum and 1% Triton X‐100, the slices were incubated with diluted primary antibodies at 4°C overnight. The primary antibodies included anti‐β‐Amyloid 1–42 antibody (Biolegend, Cat. #805509, 1:500) and anti‐AIF1/IBA1 antibody (ABclonal, Cat. #A19776, 1:1000). The fluorescence‐conjugated secondary antibodies (Jackson ImmunoResearch, 1:1000) were incubated at room temperature (RT) for 1 h. The IgG was directly detected using anti‐mouse IgG secondary antibody (1:200) at 4°C overnight. After the staining procedure was completed, the brain slice was mounted using DAPI Fluoromount‐G mounting medium (SouthernBiotech, Cat. #0100–20). Immunofluorescent images were acquired using a fluorescence microscope (ZEISS Axio Imager Z2 with ApoTome.2 optical sectioning) and analyzed with Image J software.

### 2.7 Golgi Staining

Golgi staining was performed using the FD Rapid GolgiStain Kit (FD NeuroTechnologies, Cat. #PK401). All steps were carried out according to the operation manual. Briefly, after deep anesthesia with isoflurane, the mice were decapitated to collect the brains. The brain tissues were rinsed with double‐distilled water (ddH_2_O) to clean off surface blood and then immersed into the mixture of Solution A and Solution B (1:1, prepared at least 24 h before use) for 14 days. Subsequently, the brain tissues were dehydrated in Solution C for a few days. 80 μm‐thick brain slices were cut using a vibrating microtome (Leica, 1200S). A mixture of Solution D, Solution E, and ddH_2_O (1: 1: 2) was used to develop the slices. The slices were then successively dehydrated using 50%, 75%, 95%, and 100% ethanol. Finally, after being degreased with xylene to enhance transparency, the slices were sealed with neutral balsam. The slices were photographed using a 63× oil immersion objective under bright‐field illumination. To quantify the density of dendritic spines on the apical dendrites of hippocampal CA1 region pyramidal neurons, a 30 μm segment, 50 μm away from the cell body, was analyzed. For each mouse, 20 randomly selected neurons were examined.

### 2.8 Cell Culture

The mouse hippocampal neuronal cell line HT22 cells were cultured in DMEM (high glucose) supplemented with 10% fetal bovine serum (FBS) and 1% penicillin/streptomycin (P/S) and maintained in a humidified 5% CO2 incubator at 37°C. To express human APOE3 or APOE4 in cultured cells, pcDNA3.1 expression plasmids carrying the human *APOE3* or *APOE4* gene segments were used. The plasmids and FuGene HD transfection reagent (Promega, E2311) were diluted in a 1:3 ratio using DMEM medium without FBS and P/S. After a 10‐min preincubation at room temperature, the mixture was added to the cells and incubated for 48 h at 37°C. Subsequent experiments were conducted at 48 h post‐transfection.

### 2.9 Mitochondrial Abundance Assessment

Mito‐Tracker Green (Beyotime, Cat. #C1048) was diluted in DMEM to a concentration of 100 nM to prepare the working solution. After warming up at 37°C, the working solution was used to replace the growth medium and incubated for 30 min. Following incubation, the cells were collected by centrifugation and resuspended in warm DMEM for flow cytometry analysis.

### 2.10 Mitochondrial Membrane Potential Detection

TMRM Perchlorate (MCE, Cat. #HY‐D0984A) was utilized to assess mitochondrial membrane potential. Cells were incubated with DMEM containing 100 nM TMRM Perchlorate for 1 h at 37°C. Following incubation, the cells were washed with PBS and then immediately photographed using a fluorescence microscope. The fluorescence intensity was quantified for analysis.

### 2.11 ATP Content Assay

ATP content was determined by the ATP detection kit (Beyotime, Cat. #S0026). To prepare samples for ATP assays, cells were lysed using lysis buffer on ice. After lysis, the supernatant was collected for analysis by centrifugation. The ATP detection working solution was prepared by diluting 1 volume of ATP detection reagent with nine volumes of ATP detection reagent diluent. This working solution was first added to the wells of a 96‐well black plate and incubated for 5 min to eliminate background ATP. Following this, samples were introduced into the wells, and the plate was read using a microplate reader to measure the relative light unit (RLU) value of luminescence. ATP levels were calculated based on the standard curve.

### 2.12 Reactive Oxygen Species (ROS) Measurement

To measure ROS levels, cells were incubated in DMEM containing 10 μM DCFH‐DA (Beyotime, Cat. #S0033S) for 20 min at 37°C. During incubation, cells were inverted and mixed every 5 min. after the incubation, the cells were washed with DMEM three times and then analyzed using flow cytometry.

### 2.13 Western Blotting

Total tissue protein was extracted from the hippocampus using radio immunoprecipitation assay (RIPA) lysis buffer, and cOmplete protease inhibitor cocktail tablets (Roche) were used to prevent proteolysis. Protein concentrations of the samples were determined using the BCA assay, followed by normalization with lysis buffer. After being mixed with loading buffer, the samples were boiled for 10 min at 95°C for denaturation. Protein from cultured cells was harvested directly using the loading buffer, and the cells were then scraped off and collected for boiling. Subsequently, the prepared samples were loaded onto SDS‐PAGE gels for electrophoresis. Proteins were transferred from the gel to PVDF membrane. 5% skimmed milk was used to block the membrane for 1 h at RT. Primary antibodies were incubated with the membrane overnight at 4°C, followed by horseradish peroxidase (HRP)‐conjugated secondary antibodies incubation for 1 h at RT. The blots were imaged using a chemiluminescent substrate in an imaging system (Biolight, GelView 6000). Stripping buffer (Solarbio, Cat. #SW3020) was used to remove the antibodies from the blot membrane for the incubation of new antibodies on the same membrane.

Primary antibody information: anti‐Synaptophysin antibody (Proteintech, Cat. #17785‐1‐AP, 1:20000), anti‐PSD95 antibody (Abcam, Cat. #Ab18258, 1:2000), anti‐BIG2 antibody (Proteintech, Cat. #31277‐1‐AP, 1:500), anti‐TOM20 antibody (Proteintech, Cat. #11802‐1‐AP, 1:5000), anti‐DRP1 antibody (CST, Cat. #8570, 1:1000), anti‐MFN1 antibody (CST, Cat. #14739, 1:500), anti‐MFN1 antibody (CST, Cat. #14739, 1:500), anti‐Parkin antibody (Proteintech, Cat. #14060‐1‐AP, 1:1000).

### 2.14 4D‐Label‐Free Quantitative Proteomics

Mice were thoroughly perfused with cold PBS from the heart under anesthesia. Brains were harvested to isolate the hippocampus on ice. The isolated hippocampus samples were immediately frozen in liquid nitrogen and stored in an ultra‐low temperature freezer before use. Proteins were extracted from the samples using SDT Lysis Buffer (4%(w/v) SDS, 100 mM Tris/HCl pH 7.6, 0.1 M DTT) and quantified by the BCA assay. Protein samples were digested into peptides using the filter‐aided sample preparation (FASP) method. After desalting and lyophilization, peptides were dissolved in a 0.1% formic acid solution. The prepared samples were analyzed on an LC–MS/MS platform (Bruker timsTOF Pro), which was provided by Shanghai Applied Protein Technology Co. Ltd. MaxQuant (v1.6.14) was used to analyze mass spectrometric data.

### 2.15 Label‐Free Quantitative Phosphoproteomics

Phosphopeptides were enriched by using the High‐SelectTM Fe‐NTA Phosphopeptides Enrichment Kit (ThermoFisher, Cat. #A32992) from peptide samples prepared through the FASP method. After redissolution in a 0.1% formic acid solution, the phosphopeptide samples were detected on the LC–MS/MS platform (ThermoFisher Q Exactive). Acquired raw data were analyzed using MaxQuant software.

### 2.16 Statistical Analysis

Statistical analyses were performed using GraphPad Prism 8.0. The Shapiro–Wilk test was performed to check the data normality. Comparisons between two groups were analyzed using Student's *t*‐test. One‐way ANOVA with post hoc Tukey's test was used for multiple comparisons. Two‐way repeated measures ANOVA with post hoc Tukey's test was used for multiple comparisons among the repeated measures of multiple groups over time. For data that didn't exhibit a normal distribution, the Kruskal‐Wallis test was employed for multiple comparisons. Data were presented as the means ± standard errors of the means (SEM). Statistically significant was determined by *p* < 0.05. In proteomics analysis, the differentially expressed proteins or phosphopeptides were defined as those with fold change (FC) > 2 or FC < 0.5, and a *p* < 0.05.

## 3 Results

### 3.1 Repeated Behavioral Tests Identify the Presymptomatic Stage of AD Progression in *5xFAD* Mice Expressing Human APOE3 or APOE4

**FIGURE 1:**
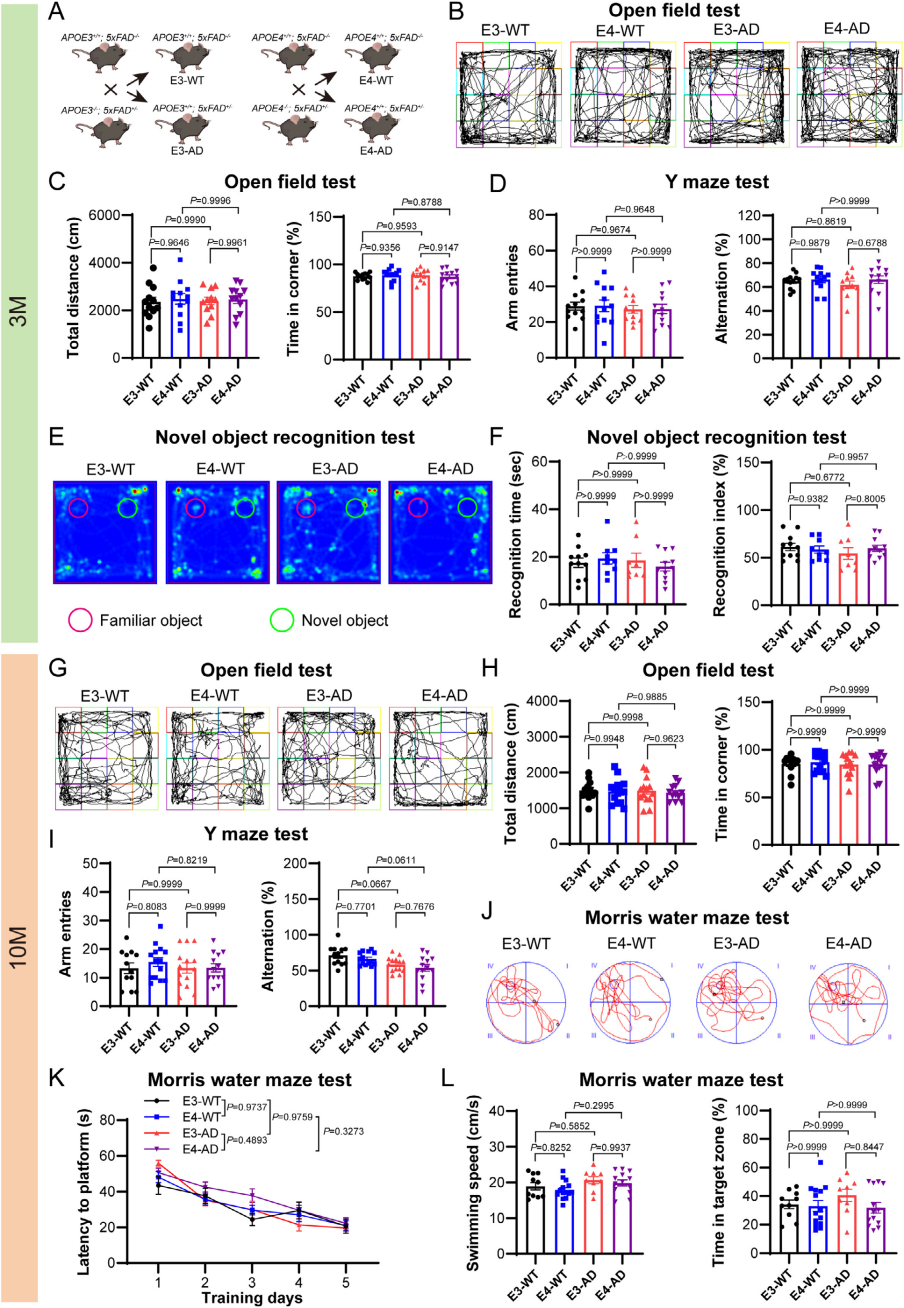
Repeated behavioral tests identify the presymptomatic stage of AD in *5xFAD* mice expressing human APOE3 or APOE4. (A) Breeding diagram of *5xFAD* mice expressing human APOE3 or APOE4 and their littermates (without *5xFAD* allele). (B–F) Behavioral tests of mice at 3‐month‐old (3 M) (*n* = 8–12 mice/group). (B) Representative images of mouse movement trajectories in the open field test. (C) Total distance of mouse movement (left panel) and the time spent in corner zone (right panel) during the open field test. (D) The number of arm entries (left panel) and the percentage of correct spontaneous alternation in Y maze test (right panel). (E) Representative heatmaps of mouse movement trajectories during novel object recognition test. (F) Recognition time (left panel) and the recognition index (right panel) during the novel object recognition test. (G–L) Behavioral tests of mice at 10‐month‐old (10 M) (*n* = 9–14 mice/group). (G) Representative track images of mouse moving during the open field test. (H) Total distance of mouse movement (left panel) and the time spent in corner zone (right panel) during the open field test. (I) The number of arm entries (left panel) and the percentage of correct spontaneous alternation in Y maze test (right panel). (J) Representative track images of mice swimming during the final test in water maze. (K) The latency of mice escaping to the platform during the training process. (L) The speed of mice during the final test in water maze (left panel) and the time spent in the quadrant zone where the platform was located (right panel). Data were analyzed using Kruskal‐Wallis test for (F) left panel, (H) right panel and (L) right panel. The data of latency to platform (K) in water maze test were analyzed using the two‐way repeated measures ANOVA. The other data were analyzed using one‐way ANOVA with post hoc Tukey's test.

To investigate whether human APOE4 accelerates the progression of AD in the presymptomatic stage, we bred the heterozygous *5xFAD* AD transgenic mice with homozygous human *APOE4* (*APOE4* ^*+/+*^; *5xFAD* ^*−/−*^, termed as E4‐WT mice) or human *APOE3* (*APOE3* ^*+/+*^; *5xFAD* ^*−/−*^, termed as E3‐WT mice) knock‐in mice to generate mice co‐expressing APOE4, APP, and PS1 genes (*APOE4* ^*+/+*^; *5xFAD* ^*+/−*^, termed as E4‐AD mice) or mice co‐expressing APOE3, APP, and PS1 genes (*APOE3* ^*+/+*^; *5xFAD* ^*+/−*^, termed as E3‐AD mice) (Figure 1A). We then conducted behavioral tests in male mice with these genotypes at 3‐month‐old age, including open field test (Figure 1B,C), Y‐maze (spontaneous alternation) test (Figure 1D), and novel object recognition test (Figure 1E,F). At this time point, neither E4‐AD mice nor E3‐AD mice demonstrated any significant behavioral differences when compared to their WT controls. In addition, E4‐AD mice did not show any significant behavioral differences compared to E3‐AD mice.

We then investigated the effects of human APOE4 versus human APOE3 on the behaviors of WT mice or *5xFAD* mice at 10‐month‐old age. We observed that neither 10‐month‐old E4‐AD mice nor 10‐month‐old E3‐AD mice demonstrated any significant behavioral differences when compared to their WT controls in the open field test (Figure 1G,H) and Y‐maze test (Figure 1I). However, we did observe that the E4‐AD mice were very close to exhibiting a significantly lower percentage of correct alternations compared to the E4‐WT mice (*p* = 0.0611). In the novel object recognition test, the 10‐month‐old *5xFAD* mice showed low interest in object exploration and very few mice met the recognition time criteria for inclusion (data not shown). Therefore, we opted to use the Morris water maze test, which is a classic cognitive function assessment experiment in AD research, as a substitute for the novel object recognition test. Previous studies have found that the *5xFAD* mice normally display cognitive defects in the Morris water maze test around 5–6 months of age [28, 29, 30]. However, the results of the Morris water maze test showed that there were no significant differences among the four groups, with the E4‐AD mice demonstrating slightly decreased time spent in the target zone compared to E4‐WT mice (Figure 1J–L).

Therefore, our comprehensive and repeated behavioral test results demonstrate that with human APOE3/4, the AD‐associated symptoms in *5xFAD* mice were very mild without statistical significance even at 10‐months‐old compared to WT controls, indicating that these mice were in the presymptomatic stage of AD.

### 3.2 Human APOE4 Causes More Deposition of Aβ Than Human APOE3 in the Brain of *5xFAD* Mice at the Presymptomatic Stage

**FIGURE 2:**
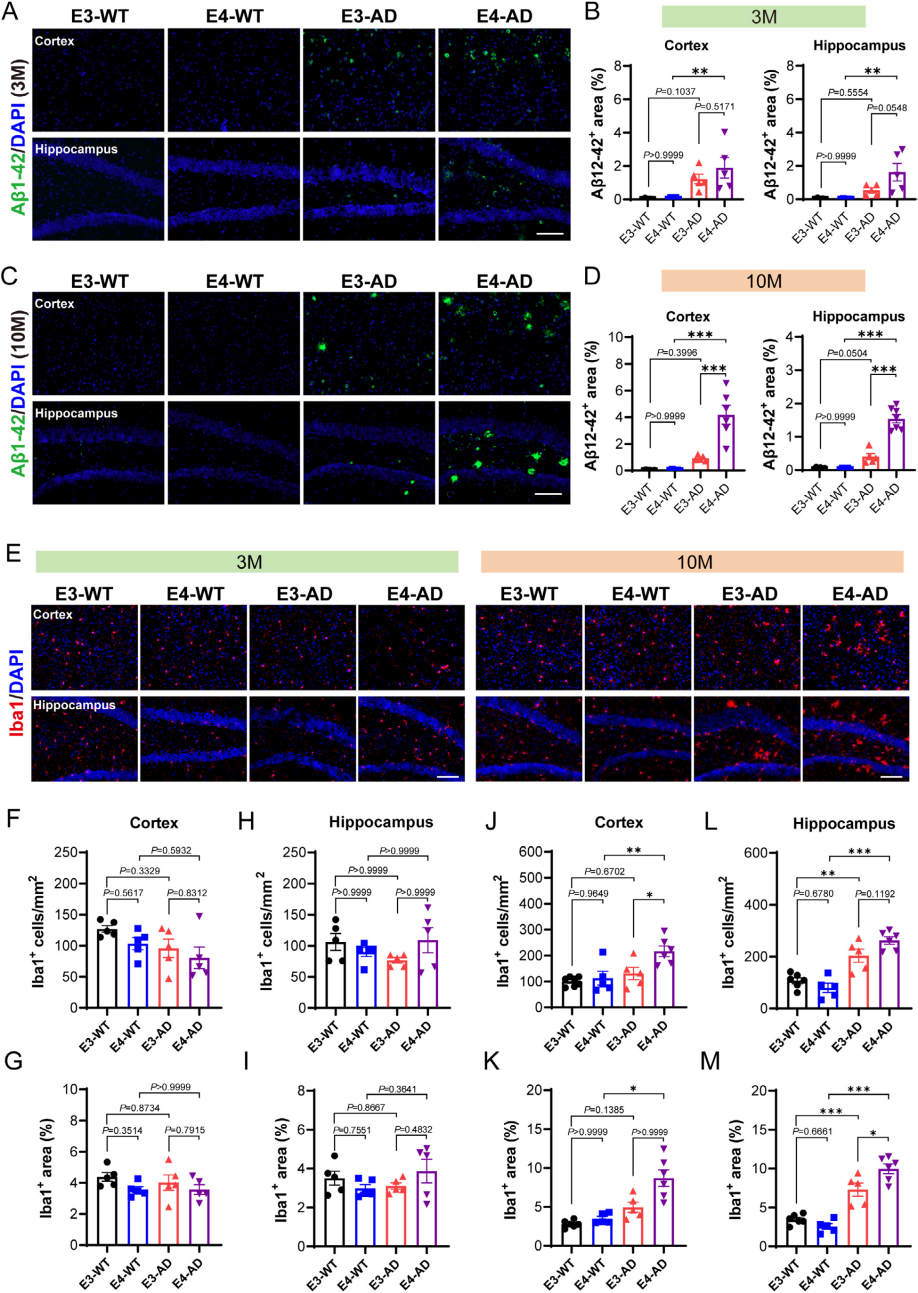
Human APOE4 causes more Aβ deposition and neuroinflammation than human APOE3 in the brain of *5xFAD* mice at the presymptomatic stage. (A, B) Representative images and quantification of immunofluorescence staining of Aβ1‐42 in the cortex and hippocampus of 3‐month‐old (3 M) mice brains. *n* = 5 mice/group. (C, D) Representative images and quantification of immunofluorescence staining of Aβ1‐42 in the cortex and hippocampus of 10‐month‐old (10 M) mice brains. (E) Representative images of immunofluorescence staining of Iba1 (a marker for microglia) in the cortex and hippocampus of 3‐month‐old (left panel) and 10‐month‐old (right panel) mouse brains. (F‐I) Quantifications of Iba1‐positive cell numbers (F, H) and Iba1‐positive cell area (G, I) in the cortex and hippocampus of 3‐month‐old mouse brains. *n* = 5 mice/group. (J‐M) Quantifications of Iba1‐positive cell numbers (J, L) and Iba1‐positive cell area (K, M) in the cortex and hippocampus of 10‐month‐old mouse brains. *n* = 5–7 mice/group. White scale scar: 50 μm, **p < 0.01, ***p < 0.001. Data were analyzed using one‐way ANOVA with post hoc Tukey's test, except for (H) and (K), which were analyzed using Kruskal‐Wallis test.

Next, we determined whether human APOE4 promotes the progression of AD at the molecular and cellular levels compared to human APOE3 in *5xFAD* mice at the presymptomatic stage. We first stained Aβ1‐42 to check amyloid pathology. At 3 months of age (Figure 2A,B), Aβ1‐42 was found in both the hippocampus and cortex of E3‐AD mice. Compared to that in E3‐AD mice, the positive area of Aβ1‐42 in the hippocampus of E4‐AD mice has a tendency to increase, with a *P* value of 0.0548. At this stage, we also observed that Aβ primarily existed not in the form of deposited plaques; it appeared to be diffusely present within the neuronal cells (Figure 2A,B). At 10 months of age (Figure 2C,D), Aβ plaques were widely present in the brains of E3‐AD and E3‐AD mice. Importantly, the amount of the Aβ plaques was significantly increased in both the hippocampus and cortex of E4‐AD mice as compared to E3‐AD mice, demonstrating that human APOE4 significantly increases Aβ pathology even before the appearance of cognitive impairment. To directly compare the progression of Aβ pathology in *5xFAD* mice with mouse APOE or with human APOE, we additionally stained the Aβ plaques in the brain of a 6‐month‐old *5xFAD* mouse (with mouse APOE gene) (Figure S1). We observed that the *5xFAD* mouse brain displayed many more Aβ plaques even at 6‐months‐old than the 10‐month‐old E3‐AD or E4‐AD mouse brains. Collectively, our data indicated that whereas human APOE can cause less Aβ pathology than mouse APOE, human APOE4 significantly aggravates the deposition of Aβ compared to human APOE3 in AD mice long before causing cognitive impairment.

### 3.3 Human APOE4 Leads to a Higher Level of Neuroinflammation Than Human APOE3 in the Brain of *5xFAD* Mice at the Presymptomatic Stage

The microglia, as the resident immune cells in the CNS, play an important role in neuroinflammation and the progression of AD [31]. We next examined microglia activation by labeling microglia with Iba1. The results showed no significant differences in the number or area of microglia among the four groups of mice at 3 months of age (Figure 2E–I), suggesting that there were no notable neuroinflammation yet at this stage in AD mice carrying human APOE. In contrast, at 10 months of age, the number and area of microglia in the hippocampus of E3‐AD mice and E4‐AD mice were both significantly higher than those in their non‐AD control mice (Figure 2E,L,M), indicating a high level of neuroinflammation in AD mice carrying human APOE at this stage. More importantly, the activation of microglia in the hippocampus of E4‐AD mice was significantly more pronounced than that in E3‐AD mice (Figure 2E,L,M). Furthermore, we noticed that whereas the microglia in the cortex of 10‐month‐old E3‐AD mice remained not activated compared to E3‐WT mice, both the number and area of microglia in the cortex of 10‐month‐old E4‐AD mice were significantly elevated compared to age‐matched E4‐WT mice (Figure 2E,J,K). These results demonstrated that human APOE4 significantly accelerates the activation of microglia and neuroinflammation in *5xFAD* AD mice at the presymptomatic stage.

The integrity of the BBB is essential to maintain the homeostasis of the CNS microenvironment and the prevention of neuroinflammation [32, 33, 34]. Therefore, we investigated whether human APOE4 accelerated neuroinflammation by increasing BBB disruption through measurement of the leakage of endogenous blood IgG in the brain. As expected, we did not observe any leakage of blood IgG in both E3‐AD and E4‐AD mice at 3 months of age (Figure S2A,B). At 10 months of age, we observed that the blood IgG level was significantly increased in the hippocampus of E3‐AD and E4‐AD mice as compared to their non‐AD control mice (Figure S2C,D), indicating that AD progression was accompanied by BBB breakdown at the presymptomatic stage. However, we did not find any significant differences in the leakage of blood IgG into the brain tissue between E4‐AD and E3‐AD mice. These data demonstrate that human APOE4 does not significantly aggravate the BBB disruption in *5xFAD* AD mice compared to human APOE3, at least during the presymptomatic stage.

### 3.4 Sequential Proteomic Analysis of Human APOE4 and APOE3‐Induced Molecular Changes in the Brain of *5xFAD* Mice at the Presymptomatic Stage

The hippocampus is one of the earliest brain regions to undergo pathological changes in AD and is central to the progression of cognitive impairments [35]. Therefore, we isolated the hippocampal tissue from E3‐AD and E4‐AD mice, as well as their non‐AD control mice, at 3 and 10 months of age, respectively, and investigated the progressive changes at the molecular level through label‐free quantitative proteomics.

**FIGURE 3:**
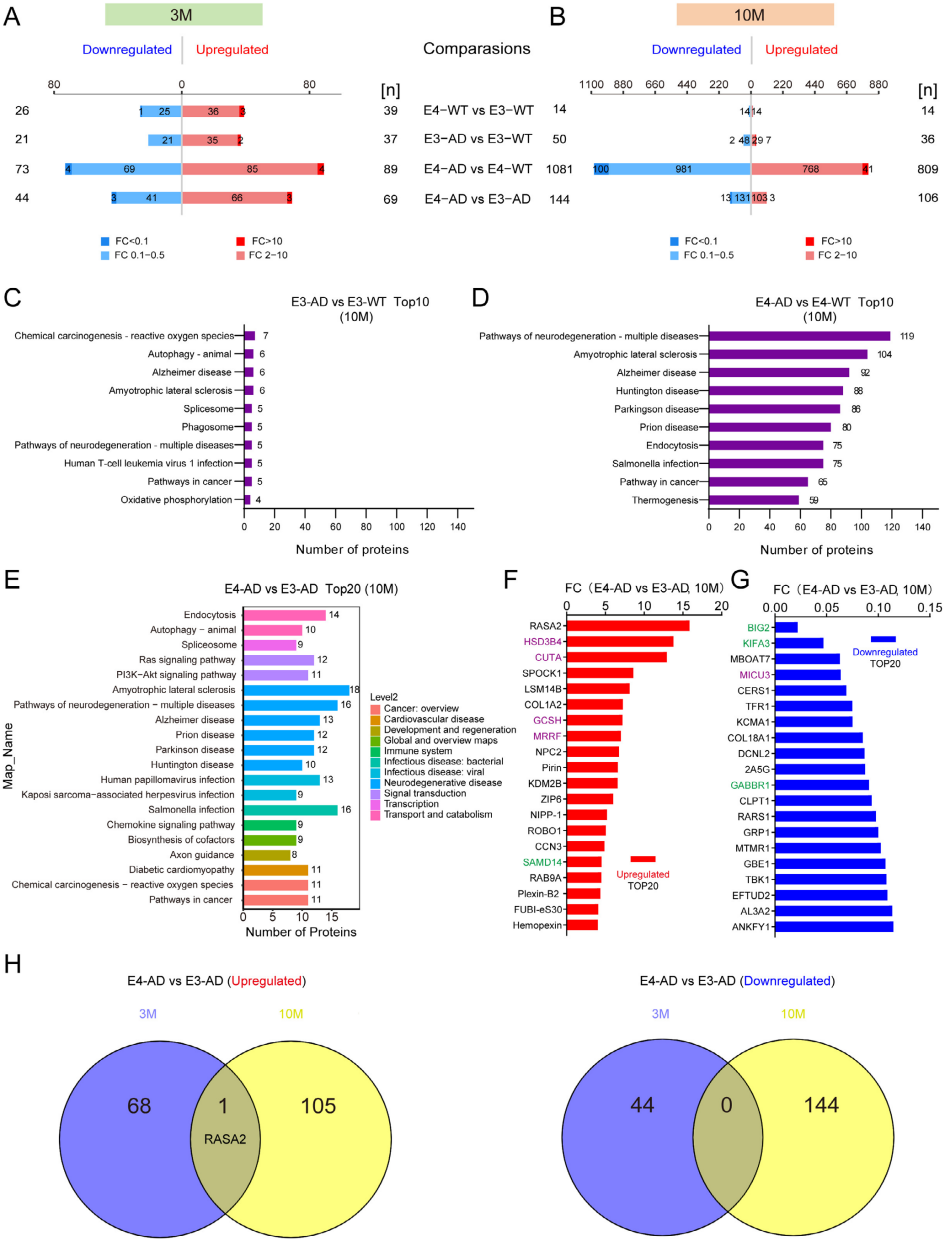
Sequential proteomic analysis of human APOE4 and APOE3‐induced molecular changes in the hippocampus of *5xFAD* mice at the presymptomatic stage. (A, B) Summary of the differentially expressed protein (DEP) numbers identified between different groups in the hippocampus of 3‐month‐old (A) and 10‐month‐old (B) mice, respectively. DEPs were defined as proteins with fold change (FC) > 2 or FC < 0.5, and a *p* < 0.05. (C, D) Top10 KEGG‐enriched pathways in comparisons between E3‐AD and E3‐WT mice (10‐month), and E4‐AD and E4‐WT mice (10‐month), respectively. (E) Top20 significantly enriched KEGG pathways at level 2 in comparisons between E4‐AD and E3‐AD mice (10‐month). (F, G) The top20 upregulated and downregulated proteins in comparisons between E4‐AD and E3‐AD mice (10‐month). Synapse‐related proteins were marked in green, while mitochondria‐related proteins were marked in purple. (H) Venn diagram showing overlap of upregulated (left panel) and (downregulated) DEPs in comparisons between E4‐AD and E3‐AD mice at the age of 3 months and 10 months, respectively.

At 3 months of age, human APOE4 induced slightly more differentially expressed proteins (DEPs) than human APOE3 in *5xFAD* mice (162 DEPs for E4‐AD vs. E4‐WT comparison and 58 DEPs for E3‐AD vs. E3‐WT comparison) (Figure 3A and Appendix S1). A more striking findings was the number of DEPs observed in 10‐month‐old *5xFAD* mice carrying human APOE4 compared to the APOE3 controls. There were 1890 proteins significantly changed in total between E4‐AD and E4‐WT mice, whereas only 86 proteins changed between E3‐AD and E3‐WT mice (Figure 3B and Appendix S2). KEGG analysis revealed that the top 6 enriched pathways associated with proteins that changed between E4‐AD and E4‐WT mice at 10 months of age were related to neurodegenerative diseases, including “Pathways of neurodegeneration‐multiple diseases”, “Amyotrophic lateral sclerosis”, “Alzheimer disease”, “Huntington disease”, “Parkinson disease”, and “Prion disease”. In the item of “Pathways of neurodegeneration‐multiple diseases” and “Alzheimer disease”, 119 and 92 proteins were enriched, respectively, while the protein numbers for E3‐AD and E3‐WT mice were only 5 and 6, respectively (Figure 3C,D). These data clearly indicated that at the molecular level, APOE4 exhibits a much stronger pathogenicity compared to APOE3 in the progression of AD.

In the comparison between E4‐AD and E3‐AD mice, 69 and 106 proteins were upregulated, and 44 and 144 proteins were downregulated in the hippocampus at 3 and 10 months of age, respectively (Figure 3A,B and Appendices S1 and S2). KEGG level 2 analysis indicated that the human diseases related DEPs also concentrated in pathways associated with neurodegenerative diseases (Figure 3E). In the signal transduction category, the Ras signaling pathway and the PI3K‐AKT signaling pathway were enriched.

In addition to Aβ deposition, neuronal APOE4 is associated with altered synaptic function and mitochondrial dysfunction [36]. Therefore, we specifically defined synaptic and mitochondrial proteins based on the subcellular location information from the UniProt database to analyze our proteomic data. Among the top 20 upregulated and top 20 downregulated differentially expressed proteins, there were 4 synaptic proteins, including SAMD14, BIG2, KIFA3, and GABBR1 (Figure 3F,G, marked with green); meanwhile, 5 mitochondrial proteins were also on the list, including HSD3B4, CUTA, GCSH, MRRF, and MICU3 (Figure 3F,G, marked with purple). We compared the DEPs between E4‐AD and E3‐AD mice at the ages of 3 and 10 months. Surprisingly, among the DEPs at 3 and 10 months of age, only one protein (RASA2) was upregulated both at 3 months and 10 months of age between E4‐AD and E3‐AD mice, whereas no protein was downregulated at both these two time points (Figure 3H). The results indicated the very different protein profiles underlying the pathogenicity of human APOE4 between 3 and 10 months of age in AD mice, and that RASA2 may play an important role in the different phases of the presymptomatic stage of AD.

### 3.5 Sequential Phosphoproteomic Analysis of Human APOE4 and APOE3‐Induced Molecular Changes in the Brain of 5xFAD Mice at the Presymptomatic Stage

**FIGURE 4:**
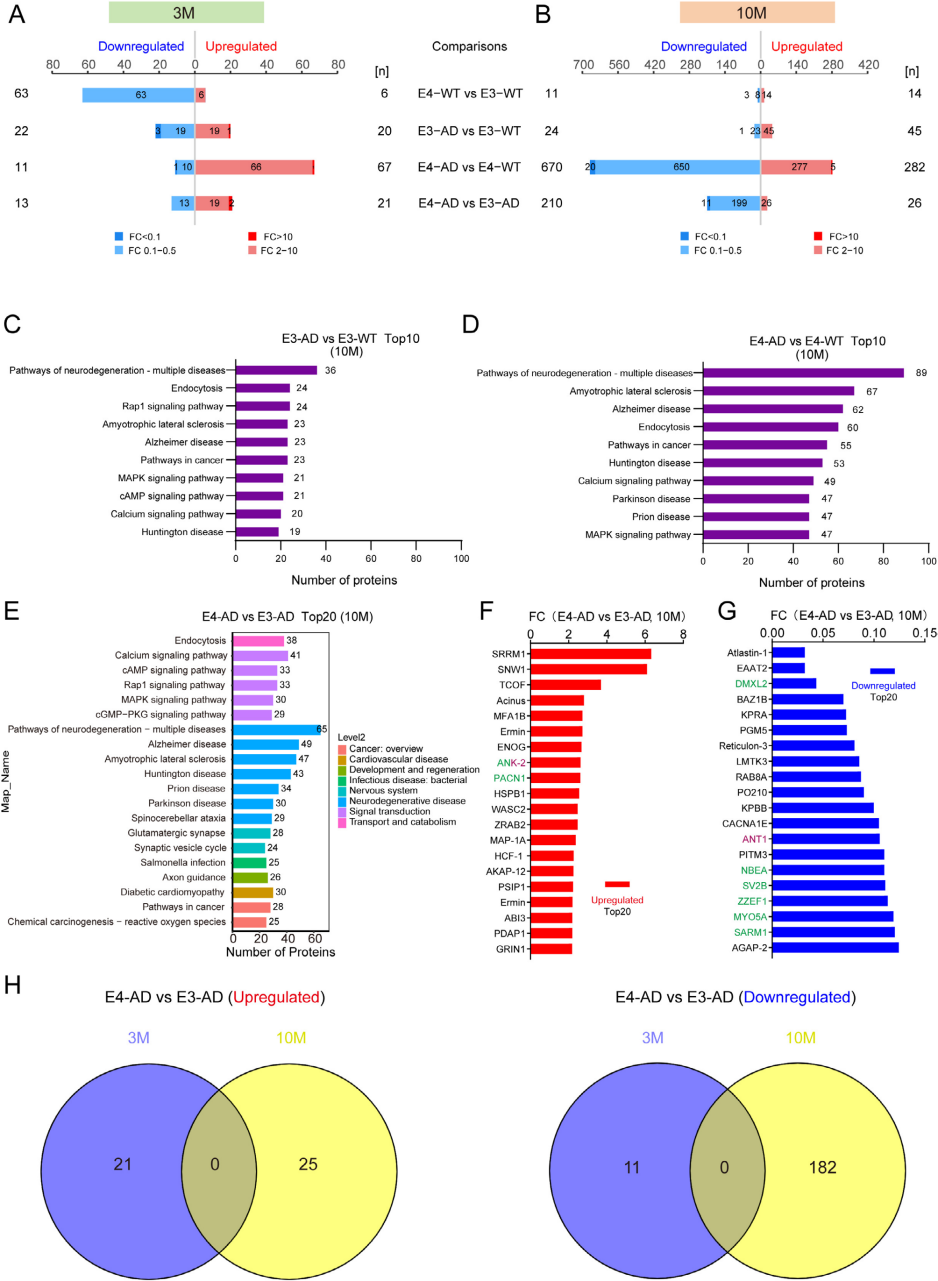
Sequential phosphoproteomic analysis of human APOE4 and APOE3‐induced molecular changes in the hippocampus of *5xFAD* mice at the presymptomatic stage. (A, B) Summary of the differentially expressed phosphopeptides (DEpP) numbers identified between different groups in the hippocampus of 3‐month‐old (A) and 10‐month‐old (B) mice, respectively. DEPs were defined as proteins with fold change (FC) > 2 or FC < 0.5, and a *p* < 0.05. (C, D) Top10 KEGG‐enriched pathways in comparisons between E3‐AD and E3‐WT mice (10‐month), and E4‐AD and E4‐WT mice (10‐month), respectively. (E) Top20 significantly enriched KEGG pathways at level 2 in comparisons between E4‐AD and E3‐AD mice (10‐month). (F, G) The top20 upregulated and downregulated phosphopeptides in comparisons between E4‐AD and E3‐AD mice (10‐month). Synapse‐related proteins were marked in green, while mitochondria‐related proteins were marked in purple. ANK‐2 was marked with two colors due to its classification as both a synapse‐related protein and a mitochondria‐related protein. A protein name may appear multiple times as different phosphopeptides from the same protein can be detected. (H) Venn diagram showing overlap of upregulated (left panel) and (downregulated) DEpPs in comparisons between E4‐AD and E3‐AD mice at the age of 3 months and 10 months, respectively.

We further investigated the effects of APOE4 on protein phosphorylation by using label‐free quantitative phosphoproteomics. Similar to changes in protein levels, human APOE4 induced slightly more differentially expressed phosphopeptides (DEpP) than human APOE3 in *5xFAD* mice at 3 months of age (78 DEpPs for E4‐AD vs. E4‐WT comparison and 42 DEpPs for E3‐AD vs. E3‐WT comparison) (Figure 4A and Appendix S3). In contrast, APOE4 induces far more protein phosphorylation changes than APOE3 at 10 months of age in AD mice, as compared to their non‐AD control mice. A total of 952 DEpPs were identified between E4‐AD and E4‐WT mice, while only 69 DEpPs were identified between E3‐AD and E3‐WT mice (Figure 4B and Appendix S4). The human diseases related pathways enriched by KEGG at 10 months of age are still mainly associated to neurodegenerative diseases between E4‐AD and E4‐WT mice. In the item of “Pathways of neurodegeneration‐multiple diseases” and “Alzheimer disease”, 89 and 62 phosphopeptides were enriched, respectively, while the numbers for E3‐AD and E3‐WT mice were 36 and 23, respectively (Figure 4C,D).

Compared to E3‐AD mice, 21 and 26 phosphopeptides were upregulated, and 13 and 210 phosphopeptides were downregulated in the hippocampus of E4‐AD mice at 3 and 10 months of age, respectively (Figure 4A,B and Appendices S3 and S4). In the top 20 items of enriched KEGG level 2 pathways (Figure 4E), 7 items are related to neurodegenerative diseases, including “Pathways of neurodegeneration‐multiple diseases”, “Alzheimer disease”, “Amyotrophic lateral sclerosis”, “Huntington disease”, “Prion disease”, “Parkinson disease”, and “Spinocerebellar ataxia”. Three pathways among the top 20 item are related to synaptic plasticity, including “glutamatergic synapse”, “synaptic vesicle cycle”, and “axon guidance”. Signaling pathways includes Calcium signaling pathway, cAMP signaling pathway, Rap1 signaling pathway, MAPK signaling pathway, and cGMP‐PKG signaling pathway.

Among the top 20 upregulated and top 20 downregulated phosphopeptides correlated proteins, eight proteins are synaptic proteins, including ANK‐2, PACN1, DMXL2, NBEA, SV2B, ZZEF1, MYO5A, and SARM1 (Figure 4F,G, marked with green). Notably, ANK‐2 is also a mitochondrial protein (Figure 4F,G, marked with purple). Besides, ANT1 is another mitochondrial protein on the list above. Between 3‐month‐old and 10‐month‐old E4‐AD and E3‐AD mice, there was no overlap in differential phosphopeptides (Figure 4H). The results suggested that APOE4 affects the progression of AD through highly distinct phosphorylation pathways in mice at 3 months and 10 months of age.

### 3.6 Human APOE4 Results in More Severe Synaptic Degeneration Than Human APOE3 in the Brain of *5xFAD* Mice at the Presymptomatic Stage

**FIGURE 5:**
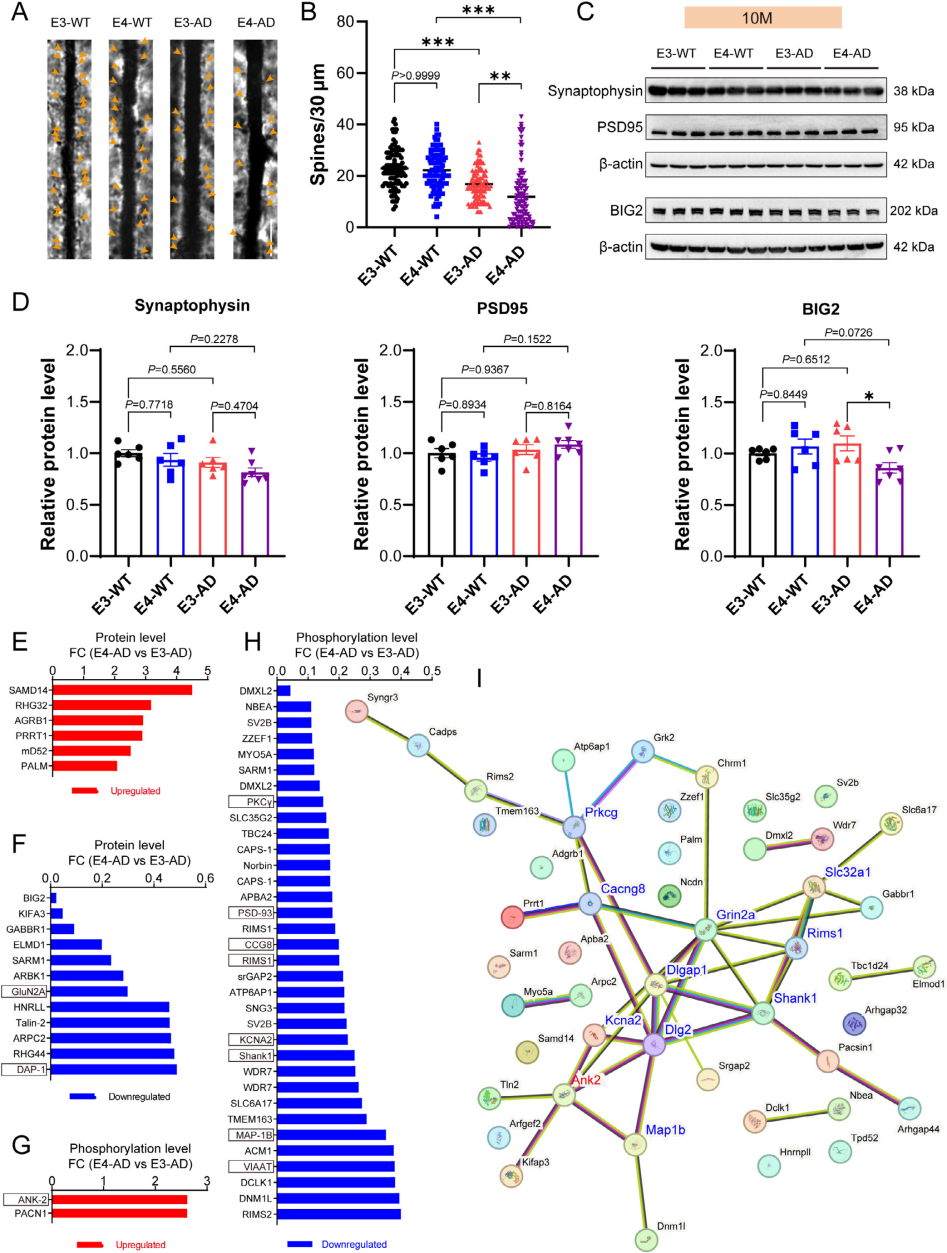
Human APOE4 results in more pronounced synaptic degeneration than human APOE3 in the brain of *5xFAD* mice at the presymptomatic stage. (A) Representative images of Golgi staining of apical dendrites (highlighted by arrows) of pyramidal neuron in the hippocampal CA1 region of 10‐month‐old mice. (B) Quantification of spines on the apical dendrites of pyramidal neuron stained in (A) Each point represents a neuron, 20 neurons per mouse, *n* = 5 mice/group. **p < 0.01, ***p < 0.001, Kruskal‐Wallis test. White scale scar: 10 μm. (C, D) Western blotting measurement of PSD95, Synaptophysin, and BIG2 expression levels. *n* = 6–7 mice/group, *p < 0.05, one‐way ANOVA with post hoc Tukey's test. (E, F) All the significantly upregulated and downregulated synapse‐related proteins in comparison between E4‐AD and E3‐AD mice (10‐month). (G, H) All the significantly upregulated and downregulated synapse‐related phosphopeptides in comparison between E4‐AD and E3‐AD mice (10‐month). A protein name may appear multiple times as different phosphopeptides from the same protein can be detected. (I) Protein–Protein Interaction (PPI) network map of all differentially expressed synapse‐related proteins and phosphopeptides in (E‐H). The hub proteins (connecting with 3 or more other proteins) identified with PPI network were marked with red (upregulated) or blue (downregulated) in the PPI network and boxed in (E‐H).

Given that the proteomic and phosphoproteomic analysis above both demonstrated robust molecular features of synaptic degeneration, we investigated the changes of synapses in the hippocampus of E3‐AD and E4‐AD mice and their controls. Golgi staining was used to analyze dendritic spines of pyramidal neurons in the hippocampus CA1 area of mice at 10 months of age (Figure 5A,B). Our data indicated that the density of dendritic spines in both E3‐AD and E4‐AD mice exhibited a significant decrease compared to their wild‐type counterparts, E3‐WT and E4‐WT. Furthermore, the density of dendritic spines in E4‐AD mice was significantly lower than that in E3‐AD mice (Figure 5A,B), indicating that APOE4 caused more synaptic degeneration than APOE3. PSD‐95 and Synaptophysin are two classic synaptic proteins usually used for assessing synaptic levels, while BIG2 is the top differentially expressed synaptic protein identified between E4‐AD and E3‐AD mice in our proteomic screening. We measured these three synaptic proteins using Western blotting to further confirm synapse loss in 10‐month E4‐AD and E3‐AD mice. Unexpectedly, the results showed that there were no significant differences in the three synaptic protein levels between E3‐AD and E3‐WT mice or between E4‐AD and E4‐WT mice (Figure 5C,D). However, the protein levels of BIG2 were found to be significantly decreased in E4‐AD mice compared to E3‐AD mice (Figure 5C,D). These data demonstrated that BIG2 can serve as a molecular marker of early synaptic degeneration that is more sensitive than classic synaptic proteins, and BIG2 may be closely involved in the molecular mechanism underlying the promoting effect of APOE4 on synapse degeneration in AD.

By combining the data of proteomics and phosphoproteomics, a total of 18 synapse‐related proteins and 36 synapse‐related phosphopeptides were identified to be significantly upregulated or downregulated between E4‐AD and E3‐AD mice at 10 months of age (Figure 5E–H). We then drew the Protein–Protein Interaction (PPI) network map of all differentially expressed synapse‐related proteins and phosphopeptides caused by APOE4 compared to APOE3 in AD mice in order to uncover the molecular basis of APOE4‐induced synaptic degeneration at the presymptomatic stage of AD (Figure 5I). As a result, 11 hub proteins (connectivity ≥ 3) were identified and marked with red (upregulated) or blue (downregulated) in the PPI network, including GluN2A (*Grin2a*), DAP‐1 (*Dlgap1*), PSD‐93 (*Dlg2*), KCNA2 (*Kcna2*), Shank1 (*Shank1*), PKCγ (*Prkcg*), VIAAT (*Slc32a1*), ANK‐2 (*Ank2*), CCG8 (*Cacng8*), RIMS1 (*Rims1*), and MAP‐1B (*Map1b*). These 11 hub proteins may individually or collaboratively act as the molecular mechanisms of APOE4‐induced synaptic degeneration in AD mice.

### 3.7 Human APOE4 Impairs Neuronal Mitochondrial Function in the Brain of *5xFAD* Mice at the Presymptomatic Stage

**FIGURE 6:**
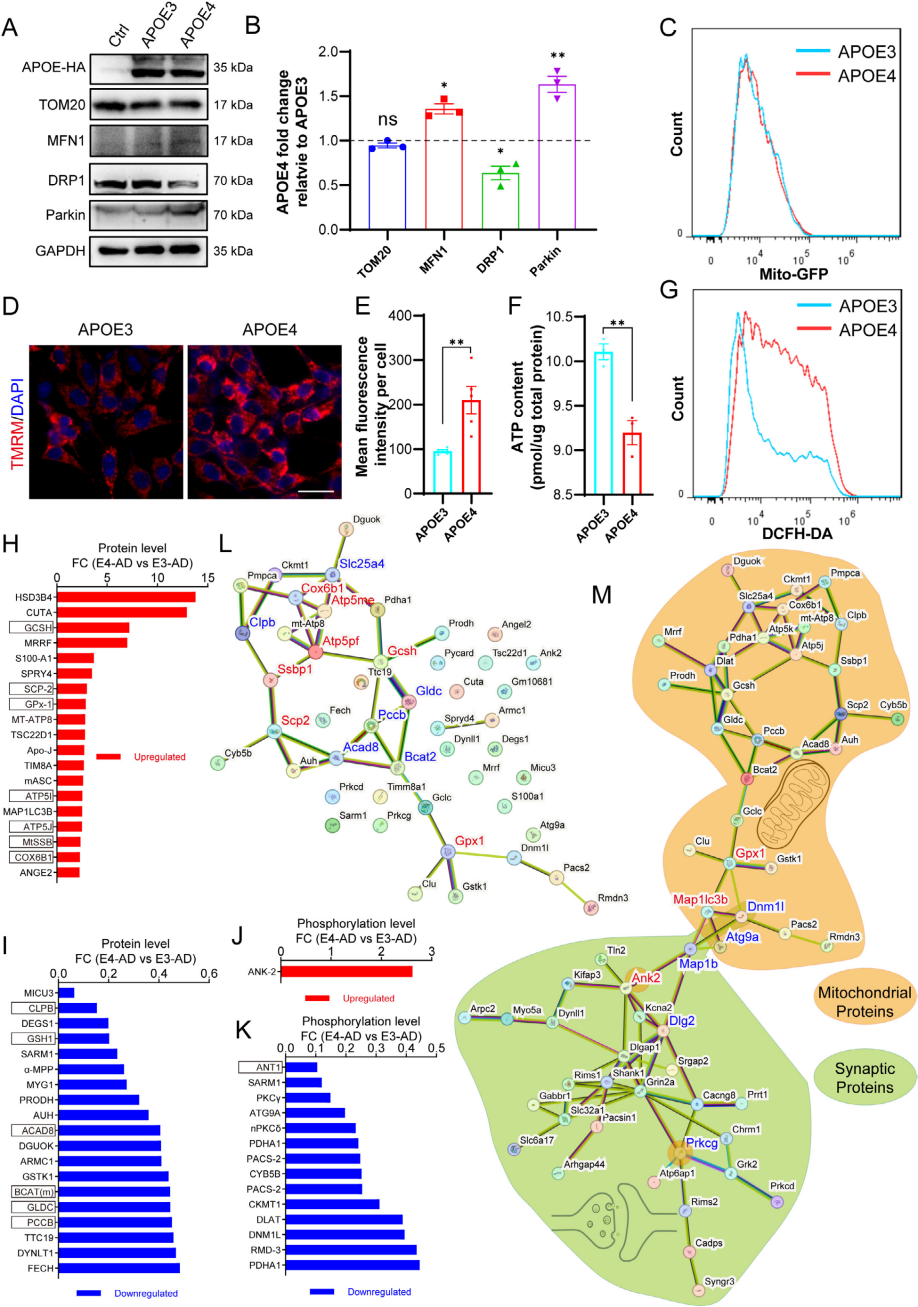
Human APOE4 impairs neuronal mitochondrial function in the brain of *5xFAD* mice at the presymptomatic stage. (A, B) Western blotting measurement of mitochondrial function‐related protein expressions, including TOM20, MFN1, DRP1, Parkin, in mouse hippocampal neuronal cell line HT22 cells expressing human APOE3 or APOE4 fused to HA tag. GAPDH served as an internal control. (C) HT22 cells incubated with Mito‐GFP were analyzed by flow cytometry to determine mitochondrial content. (D, E) Representative images and quantification of mitochondrial membrane potential with TMRM. (F) ATP content measured by firefly luciferase. (G) HT22 cells incubated with DCFH‐DA were analyzed by flow cytometry to determine ROS levels. *n* = 3–5 biological replicates per treatment. *p < 0.05, **p < 0.01, Student's *t*‐test. (H, I) Significantly upregulated and downregulated mitochondria‐related proteins in comparison between E4‐AD and E3‐AD mice (10‐month). (J, K) Significantly upregulated and downregulated mitochondria‐related phosphopeptides in comparison between E4‐AD and E3‐AD mice (10‐month). A protein name may appear multiple times as different phosphopeptides from the same protein can be detected. (L) Protein–Protein Interaction (PPI) network map of all differentially expressed mitochondria‐related proteins and phosphopeptides in (H‐K). The hub proteins (connecting with 3 or more other proteins) identified with PPI network were marked with red (upregulated) or blue (downregulated) in the PPI network and boxed in (H‐K). (M) PPI network map of all differentially expressed proteins and phosphopeptides associated with mitochondria and synapse. Proteins in the key nodes were determined with having direct connection to synaptic proteins PPI network or mitochondrial proteins PPI network rather than having connection to a single protein. The gene names in the key nodes marked with red or blue means upregulated or downregulated in protein or phosphopeptides.

To determine the direct effects of APOE4 versus APOE3 on neuronal mitochondrial function, we constructed pcDNA3.1 plasmids encoding human APOE3 or APOE4 and transfected them into the mouse hippocampal neuronal cell line HT22 cells to express APOE3 or APOE4. Our Western blotting results confirmed the successful expression of APOE3 and APOE4 in HT22 cells, with their expression levels being equivalent (Figure 6A). Next, we examined the expression levels of several key proteins associated with mitochondrial dynamics and function: mitochondrial marker TOM20, mitochondrial fusion marker MFN1, mitochondrial fission marker DRP1, and mitophagy marker Parkin. Our results revealed that in HT22 cells expressing APOE4, the expressions of TOM20 were not changed, MFN1 and Parkin were upregulated, while DRP1 was downregulated, as compared to those in HT22 cells expressing APOE3 (Figure 6A,B). These observations suggested that APOE4 disrupts mitochondrial homeostasis in HT22 cells by promoting mitochondrial fusion, increasing mitophagy, and suppressing mitochondrial fission. There was no significant change in TOM20 expression (Figure 6A,B), indicating that APOE4 did not alter the quantity of mitochondria in HT22 cells. This was further confirmed by detecting the mitochondrial tracer Mito‐GFP using flow cytometry (Figure 6C). Compared to HT22 cells expressing APOE3, those expressing APOE4 exhibited significantly increased membrane potential (Figure 6D,E), a change that promotes mitochondrial fusion and is consistent with the observed upregulation of MFN1 expression. We further examined mitochondrial function by measuring ATP content and reactive oxygen species (ROS) levels in these cells. We observed that the expression of APOE4 resulted in a significant reduction in ATP levels and a significant elevation in ROS levels within the HT22 cells (Figure 6F,G). The results were indicative of mitochondrial dysfunction and provided strong evidence for the direct adverse impact of APOE4 on mitochondrial function compared to APOE3. Collectively, our data demonstrate that APOE4 causes mitochondrial dysfunction by promoting mitochondrial fusion and mitophagy while inhibiting mitochondrial fission, rather than by altering the quantity of mitochondria.

We subsequently analyzed our proteomic data to identify the key proteins and molecular mechanisms implicated in mitochondrial dysfunction during AD development in mice carrying human APOE4. There were 38 mitochondria‐related proteins and 15 mitochondria‐related phosphopeptides identified to be significantly upregulated or downregulated between E4‐AD and E3‐AD mice at 10 months of age (Figure 6H–K). With the genes of these proteins and phosphopeptides related proteins, we created the Protein–Protein Interaction (PPI) network map of all differentially expressed mitochondria‐related proteins and phosphopeptides caused by APOE4 compared to APOE3 in AD mice in order to uncover the molecular basis of APOE4‐induced mitochondrial impairment at the presymptomatic stage of AD (Figure 6L). As a result, 13 hub proteins (connectivity ≥ 3) were identified and marked with red (upregulated) or blue (downregulated) in the PPI network. The 13 hub proteins included ATP5J (*Atp5pf*), GCSH (*Gcsh*), ANT1 (*Slc25a4*), ACAD8 (*Acad8*), ATP5I (*Atp5me*), BCAT(m) (*Bcat2*), COX6B1 (*Cox6b1*), GPx‐1 (*Gpx1*), PCCB (*Pccb*), SCP‐2 (*Scp2*), CLPB (*Clpb*), GLDC (*Gldc*), and MtSSB (*Ssbp1*). These 13 proteins may individually or collaboratively act as the molecular mechanisms of APOE4‐induced mitochondrial function impairment in AD mice.

To further elucidate the connection of mitochondrial dysfunction with synaptic degeneration, we constructed a Protein–Protein Interaction (PPI) network map incorporating all differentially expressed mitochondrial and synaptic proteins, as well as differential phosphopeptides related to mitochondria and synapse. As shown in Figure 6M, in which scattered genes have been removed, there was a bridge connection between the mitochondrial protein network map (with orange background) and the synaptic protein network map (with green background). Seven proteins, GPx‐1 (*Gpx1*), MAP1LC3B (*Map1lc3b*), DNM1L (*Dnm1l*), ATG9A (*Atg9a*), MAP1B (*Map1b*), ANK‐2 (*Ank2*) and PSD‐93 (*Dlg2*) were positioned as the key nodes of the “bridge” Additionally, ANK‐2 (*Ank2*) and PKCγ (*Prkcg*), which are both mitochondrial and synaptic proteins, occupied pivotal positions in the synaptic protein network and may be involved in mitochondrial dysfunction‐mediated synaptic degeneration in E4‐AD mice. These data revealed a close interaction between mitochondrial dysfunction and synaptic degeneration in the pathological changes caused by APOE4 at the presymptomatic stage of AD and identified the seven node proteins mentioned above as candidate molecular mechanisms for further investigation.

## 4 Discussion

Longitudinal multimodal biomarker studies reveal that the AD continuum has a long preclinical stage, during which the AD pathophysiological cascade develops silently [37]. APOE4, which was previously considered the strongest genetic risk factor for sporadic AD, has recently been proposed as a distinct genetic form of AD [22]. Despite a wealth of research based on clinical biological markers continually highlighting the importance of APOE4 in AD, our understanding of the pathological mechanisms by which APOE4 promotes the progression of AD remains fragmented. In this study, we conducted a comprehensive analysis of the cellular and molecular effects of APOE4 compared to APOE3 on brain tissue and neurological function in humanized AD mouse models during the presymptomatic stage. We further mapped the proteomic and phosphoproteomic changes in the hippocampus induced by APOE4 versus APOE3. Our findings indicate that APOE4 accelerates AD pathologies in *5xFAD* mice at the presymptomatic stage, affecting Aβ deposition and microglial activation but not BBB disruption. Proteomics analyses revealed that APOE4 significantly enhances the activity of neurodegeneration and AD‐related pathways during this stage. Importantly, we identified robust molecular features of synaptic degeneration and mitochondrial dysfunction induced by APOE4, drew three Protein–Protein Interaction network maps for the mitochondrial impairment and synaptic degeneration caused by APOE4 in AD mice, and discovered a close interaction between these processes and the key proteins that mediated this interaction. Lastly, to elucidate the molecular mechanisms of APOE4‐induced mitochondrial impairment and synaptic degeneration in AD, we identified over 10 pivotal proteins (RASA2, BIG2, GPx‐1, MAP1LC3B, DNM1L, ATG9A, MAP1B, ANK‐2, PSD‐93, PKCγ, etc.) and several signaling pathways (Ras signaling pathway, PI3K‐AKT signaling pathway, Calcium signaling pathway, cAMP signaling pathway, Rap1 signaling pathway, etc.) that are significantly regulated by APOE4 and warrant further investigation. These insights deepen our understanding of the pathogenic role of APOE4 in AD and provide potential therapeutic targets for early intervention.

To determine the prosymptomatic stage of *5xFAD* mice carrying human APOE, a series of behavioral tests were used to assess the cognitive functions of mice in this study. Our data show that neither E4‐AD mice nor E3‐AD mice demonstrated any significant behavioral differences when compared to their WT controls until 10 months of age. This finding seems unexpected, as most previous studies report cognitive impairments detectable by the water maze in *5xFAD* mice after 5–6 months of age [28, 29, 30]. Since the *5xFAD* mouse model is generated by knocking in human APP along with PS1 to overexpress Aβ [38], Aβ pathology is the cause of AD‐like symptoms in *5xFAD* mice. However, we observed that even in 10‐month‐old E4‐AD mice, the Aβ plaques in the brain were fewer than those in 6‐month‐old normal (mouse APOE) *5xFAD* mice. Previous reports have indicated that human APOE‐targeted replacement can decrease Aβ deposition in *5xFAD* and APPV717F mice [39, 40]. That suggests that human APOE is less conducive to Aβ deposition compared to mouse APOE. Despite this, our results showed that human APOE4 significantly promotes Aβ deposition compared to APOE3 at the early stage of AD. The reduction of Aβ deposition by human APOE‐targeted replacement also contributes to extending the asymptomatic phase of *5xFAD* mice. Our behavioral results indicate that 10‐month‐old E3‐AD mice have not yet exhibited significant cognitive impairments, whereas E4‐AD mice at the same age are inclined to demonstrate cognitive deficits in the Y‐maze test, suggesting that APOE4 accelerates the progression of AD. Therefore, 3‐month‐old and 10‐month‐old E3‐AD and E4‐AD mice in this study effectively model the different phases of the preclinical stage of AD.

Our sequential proteomics data at both 3 months of age and 10 months of age show that APOE4‐induced protein and protein phosphorylation changes are substantially associated with neurodegenerative diseases, providing molecular evidence for the high pathogenicity of APOE4 versus APOE3. The strong promotion of AD by APOE4 is well‐documented, especially with a clinical study this year showing that almost all APOE4 homozygotes eventually develop AD, leading to APOE4 being considered a distinct genetic form of AD [22]. However, the earliest molecular events caused by APOE4 remain poorly defined. We found that among the differential proteins and phosphopeptides, RASA2 is the only one that was significantly upregulated in both 3‐month‐old and 10‐month‐old E4‐AD mice, compared to E3‐AD mice. RASA2 is a negative regulator of RAS signaling. The RAS family of GTPases is active in their GTP‐bound state, enabling them to bind to and activate downstream effectors. RASA2 activates the GTPase enzyme activity of RAS to hydrolyze GTP bound to RAS into GDP, thereby inactivating the RAS signaling [41]. Importantly, in our KEGG analysis, the RAS signaling pathway is the most enriched among the differentially expressed proteins. RAS signaling serves as the central node of a wide range of signaling pathways involved in cell survival, proliferation, differentiation, migration, and adhesion [42]. The RasGRF1 (an activator of RAS signaling) and RAS/MAPK signaling axis regulate dendritic spine development and synapse formation [43, 44, 45], suggesting the inactivation of RAS signaling may contribute to synapse loss in AD. Meanwhile, activation of RAS GTPase and its downstream MAPK pathway were also proven to promote Tau hyperphosphorylation and amyloid pathology [46]. Current research on the role of RAS signaling in AD is not sufficient and warrants further investigation. In addition to RAS signaling, the PI3K‐AKT signaling pathway, Calcium signaling pathway, cAMP signaling pathway, Rap1 signaling pathway, MAPK signaling pathway, and cGMP‐PKG signaling pathway also stand out in our proteomic analysis, suggesting their potentially important roles in the pathogenesis of APOE4 in AD.

The exacerbation of synaptic degeneration represents the decrease in neural plasticity, which is the foundation for cognitive impairment in AD. Our data demonstrate that APOE4 already caused more significant synaptic degeneration than APOE3 at the presymptomatic stage in *5xFAD* AD mice, a point evidenced by our detection of dendritic spines in the hippocampus. Our proteomics data further indicate that APOE4 causes changes in a host of synaptic proteins, including alterations at both the protein level and the phosphorylation level. These changes may be the molecular mechanisms underlying APOE4‐induced degeneration in synaptic structure and function. For instance, BIG2, KIFA3, and GABBR1 are the three synaptic proteins among the top 20 downregulated proteins induced by APOE4 in AD mice. BIG2 has been reported to regulate dendritic Golgi polarization and dendrite growth through ARF1‐RhoA‐mDia1 signaling [47]. The KIFA3 is associated with axonal polarity, playing a crucial role in axonal development [48]. The metabotropic GABAB receptors, which are essential components of neuronal inhibition in the brain, form heterodimers consisting of the GABBR1 and GABBR2 subunits [49]. These receptors contribute to the modulation of synaptic activity by mediating inhibitory signals within the central nervous system. The imbalance between excitatory and inhibitory synapses is considered a significant cause of abnormal cognitive functions and other related processes in AD [50]. GluN2A (*Grin2a*) is another synaptic protein downregulated in E4‐AD mice. A previous study reported that APOE4 promotes the loss of mature dendritic spines, which is associated with GluN2A in the dendritic spines [51]. Our PPI analysis indicated that GluN2A had the highest degree of connectivity with other proteins in the synaptic protein PPI network, suggesting a key role for GluN2A in APOE4‐induced synapse degeneration in AD. Additionally, we have observed that the changes in synaptic proteins are more attributable to a decrease in protein phosphorylation levels rather than a decrease in protein levels, highlighting a more important role of protein phosphorylation changes in APOE4‐induced synaptic degeneration. Our results also show that the levels of classic synaptic proteins such as PSD95 and synaptophysin are not significantly changed. This finding indicates that classic synaptic proteins are not altered by APOE4 at the presymptomatic stage of AD. Instead, the synaptic proteins identified by our study, such as BIG2, can serve as a more sensitive biomarker for early synaptic degeneration. In addition, we speculate that the phosphorylation of synaptic proteins may play a major role in the early morphological changes of synapses induced by APOE4 in AD. Our KEGG results also support our speculation since items related to synapses enriched in differential phosphopeptides‐associated proteins are more prominent than those in differential proteins.

The pathogenic mechanisms of APOE4 in AD can be divided into Aβ‐related and Aβ‐independent mechanisms [52]. Our cell culture experiments demonstrate that APOE4 can exert directly adverse effects on mitochondrial homeostasis in neurons, such as promoting mitochondrial fusion, increasing mitophagy, and suppressing mitochondrial fission without affecting the quantity of mitochondria. Mitochondria are highly dynamic organelles, adjusting their distribution through fission and fusion to meet the energy demands of various cellular processes [53]. Synaptic activities, such as vesicle release and reuptake, are highly dependent on the energy supply provided by mitochondria located at the synapses [54]. The reduction of synaptic mitochondria can quickly affect synaptic plasticity [55]. Our results show that in addition to the homeostasis of mitochondria, the function of mitochondria is also impaired by APOE4, including reduced energy supply and increased ROS production. The impact of APOE4 on both mitochondrial homeostasis and function can be detrimental to the maintenance of synaptic plasticity. We identified several key mitochondrial proteins that may be involved in mitochondrial function impairment and synaptic degeneration caused by APOE4 through PPI network analysis, including GPx‐1, MAP1LC3B, DNM1L, ATG9A, MAP1B, ANK‐2, and PKCγ. But we cannot be certain that these changes in proteins or protein phosphorylations are necessarily harmful. For example, GPx‐1, which was found to be upregulated in our study, possesses antioxidant properties [56]. However, some studies have indicated that overexpression of GPx‐1 promotes protein misfolding due to the reduction in the necessary ROS levels [57]. In other words, the alterations in protein expression or modification observed in this study could potentially be the precipitating factor for the exacerbation of AD pathogenesis. Alternatively, these changes might represent endogenous reparative responses that were upregulated in conjunction with the advancing pathology. Further research is warranted to elucidate their functional roles in the pathogenicity of APOE4 in AD.

Limited by the availability of antibodies, especially phosphorylation‐specific antibodies, this study was not able to perform adequate validation for most of the differential proteins and protein phosphorylation identified by proteomics. Besides, APOE4 has diverse roles in AD pathogenesis in different brain cell types, including astrocytes, neurons, microglia, oligodendrocytes, and vascular cells [58]. Further research on the proteomics results should also take into account the cellular specificity of these proteins and pathways.

Overall, our study provides a comprehensive elucidation of the pathological processes and molecular changes induced by APOE4 at the presymptomatic stage of AD. The sequential proteomic database generated in this study offers valuable mechanistic insights into the pathogenesis of AD and serves as a crucial resource for identifying new therapeutic targets and developing novel drugs for the treatment of AD.

## Author Contributions

J.C. and P.W. planned and designed the experiments. P.W. performed the animal experiments, analyzed the data, and wrote the manuscript. K.L. participated in the animal experiments, analyzed the data, and wrote the manuscript. X.C. participated in the data analysis and wrote the manuscript. C.F. and L.Q. assisted with the animal and cell culture experiments as well as data analysis. J.C. and J.H. conceived and supervised the project, interpreted the data, and wrote the manuscript.

## Conflicts of Interest

The authors declare no conflicts of interest.

## Supporting information


Figures S1–S2.

Appendix S1.

Appendix S2.

Appendix S3.

Appendix S4.

## Data Availability

All data presented in this study are either included in this article and its Figures S1 and S2 and Appendices [Link], [Link] or is available from the corresponding author upon reasonable request.
