# Supplementary material for: Sequential Proteomic Analysis Reveals the Key APOE4‐Induced Pathological and Molecular Features at the Presymptomatic Stage in Alzheimer's Disease Mice
Source: CNS Neurosci Ther. 2025 Mar 12;31(3):e70306. doi: 10.1111/cns.70306 (PMC11903334; doi:10.1111/cns.70306)
Supplement: Supplementary file 1 — Figures S1–S2. [file CNS-31-e70306-s002.pdf]

## Supplementary Figures

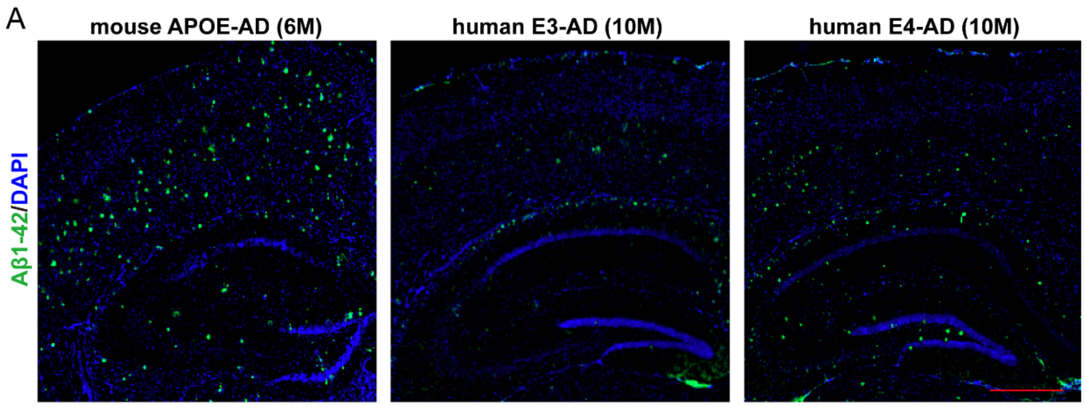


**Figure S1. Human APOE3 and APOE4 causes less Aβ deposition than mouse APOE in the brain of *5xFAD* mice.**

Representative images of immunofluorescence staining of Aβ1-42 in the brains of 6-month-old (6M) *5xFAD* mice expressing the endogenous mouse APOE and 10-month-old (10M) E3-AD and E4-AD mice. red scale scar: 500 μm

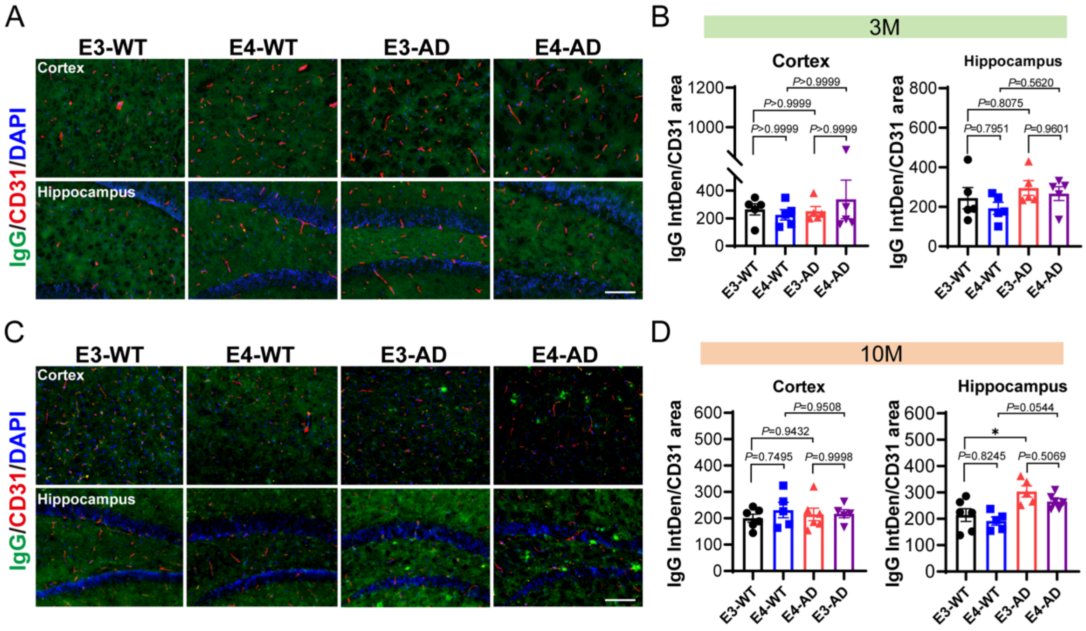


**Figure S2. Human APOE4 makes no differences in BBB disruption compared to human APOE3 in the brain of *5xFAD* mice at the presymptomatic stage.**

**(A-B)** Representative images and quantification of co-immunofluorescence staining of endogenous plasma IgG with CD31 (a marker for endothelial cells) in the cortex and hippocampus of 3-month-old mouse brains. n=5 mice/group. **(C-D)** Representative images and quantification of co-immunofluorescence staining of endogenous plasma IgG with CD31 in the cortex and hippocampus of 10-month-old mouse brains. n=5-6 mice/group. White scale scar: 50 µm. ∗ $P$<0.05, data of (**B**) left panel were analyzed using Kruskal-Wallis test, the others were analyzed using one-way ANOVA with post hoc Tukey's test.
